# A Rare Case of Streptococcus pneumoniae Complicated With Pericardial Abscess

**DOI:** 10.7759/cureus.47780

**Published:** 2023-10-27

**Authors:** Rand Sabanci, Khalid Saeed Al-Asad, Moiz Saaed, Andrew Geunwon Kim, Adolfo Martinez, Sandeep Banga, George Abela

**Affiliations:** 1 Internal Medicine, Michigan State University, East Lansing, USA; 2 Cardiology, Michigan State University, Lansing, USA; 3 Cardiology, West Virginia University School of Medicine, Morgantown, USA; 4 Cardiology, Michigan State University, East Lansing, USA

**Keywords:** streptococcus pneumoniea, community aquired pneumonia, loculated pleural effusion, acute pericardial effusion, pericardial tamponde

## Abstract

This abstract presents the case of a 37-year-old female with no significant past medical history who presented to the emergency department with a unique and challenging clinical scenario. The patient complained of chest pain, dyspnea, and a productive cough associated with stabbing chest pain that improved with leaning forward for the past week. Despite an initial diagnosis of community-acquired pneumonia, the patient's condition deteriorated rapidly, leading to septic shock. Blood cultures ultimately revealed Streptococcus pneumoniae as the causative organism. Subsequent imaging and diagnostic procedures demonstrated a complex clinical course, including loculated pleural and pericardial effusions. The patient's condition necessitated multiple interventions, including pericardiocentesis, chest tube placement, and intracavitary lytic therapies, in addition to intubation for acute respiratory failure.

The case further evolved with the development of a pericardial abscess, successfully managed with surgical drainage and a partial pericardiectomy. The patient eventually showed significant clinical improvement and was discharged on a targeted antibiotic regimen. This case highlights the importance of vigilance in identifying rare complications of pneumonia and the need for prompt, multidisciplinary management to ensure the best possible outcome for the patient. Long-term follow-up was recommended to assess the patient's recovery. This case underscores the complexities and challenges of managing uncommon presentations of infectious diseases and emphasizes the value of a comprehensive, multidisciplinary approach in such cases.

## Introduction

Purulent pericarditis is a serious complication of pneumonia. Around 40-50% of all cases of purulent pericarditis are caused by Gram-positive bacteria, particularly *Streptococcus pneumoniae* [1]. Over the past 70 years, however, it has become largely eliminated following the introduction of antibiotics in the 1940s and with the addition of the pneumococcal conjugate vaccine to vaccination regimens [1]. Herein, we present a case of primary *S. pneumoniae* pericarditis that developed in an immunocompetent 37-year-old female patient. The patient had a protracted ICU course complicated by cardiac tamponade secondary to a huge pericardial abscess and multiple empyemas.

## Case presentation

A 37-year-old female patient with no significant past medical history presented to the emergency department with a complaint of chest pain. The patient described stabbing chest pain that improved with leaning forward for the past week. She had associated dyspnea and a persistent cough with green sputum. She denied any fever, chills, or recent infection.

On presentation, she was afebrile, tachycardic, and tachypneic, with normal oxygen saturation in room air. On a physical exam, she appeared lethargic and pale, had normal heart sounds, diminished air entry to the left lung with dull percussion, and increased tactile fremitus in the left lower lobe. The rest of the physical exam was unremarkable.

**Figure 1:**
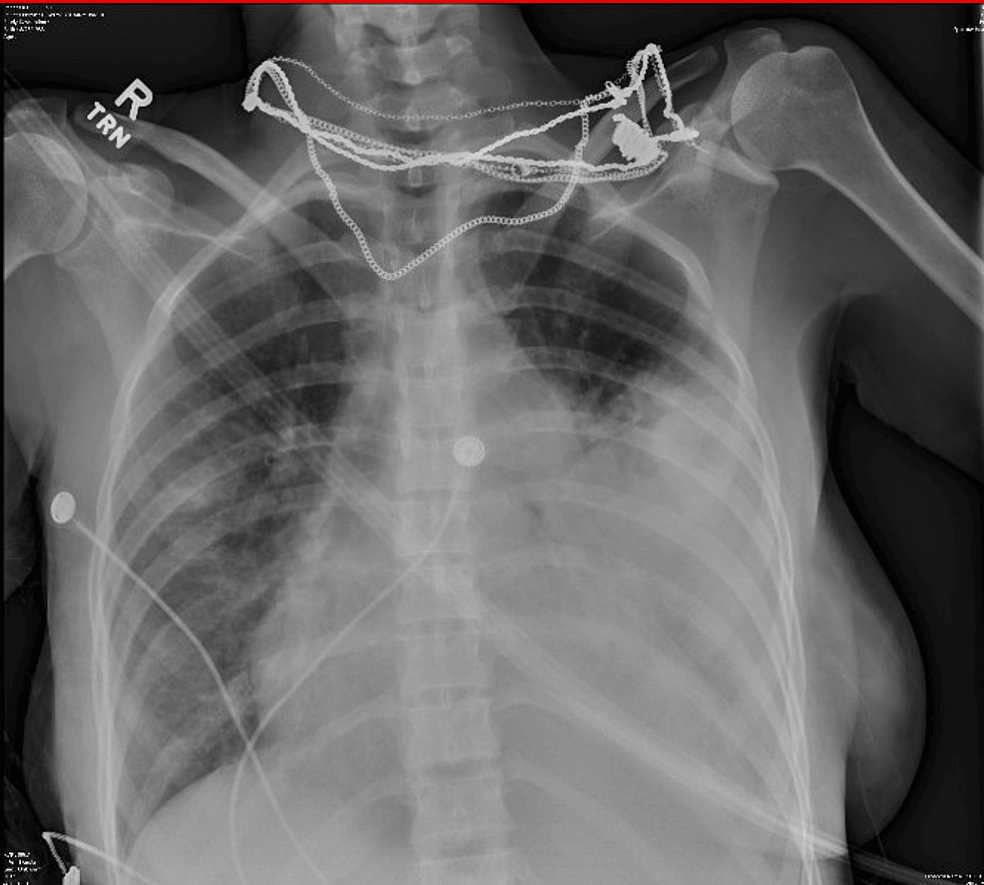
Initial chest X-ray showing cardiomegaly, patchy infiltrates in the right mid-lung and lung base with extensive consolidation in the left mid-lung, lung base, and a left pleural effusion.

**Figure 2:**
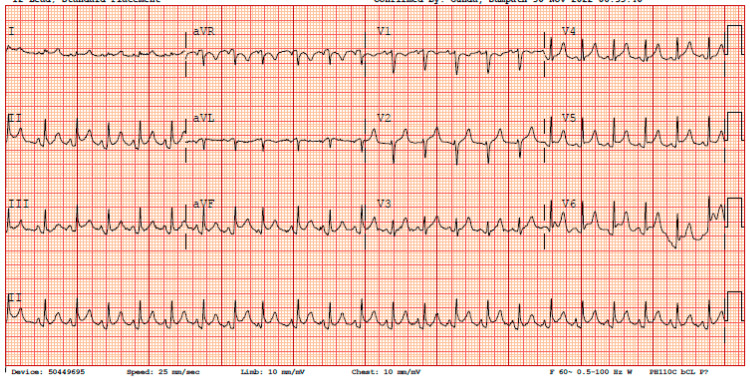
Initial EKG showing sinus tachycardia with diffuse concave ST segment elevation and PR depression suggestive of acute pericarditis.

Her initial laboratory workup revealed leukocytosis of 30 × 10^3^ with a left shift, elevated C-reactive protein of 12 mg/L, and elevated lactate (2.8 mmol/L). The initial chest X-ray showed cardiomegaly, patchy infiltrates in the right midlung and base, and extensive consolidation in the left midlung extending to the lung base, with blunting of the left costophrenic angle suggestive of pleural effusion, which can be appreciated in Figure 1. The electrocardiogram (EKG) showed diffuse ST segment elevation with PR depression, as seen in Figure 2.

The patient was admitted for septic shock with suspicion of community-acquired pneumonia as the primary source of infection and was treated with intravenous (IV) fluid hydration and broad-spectrum antibiotics, including IV vancomycin, cefepime, and metronidazole. Despite these measures, she became hypotensive and required norepinephrine. Ibuprofen and colchicine were also initiated for suspected pericarditis.

**Figure 3:**
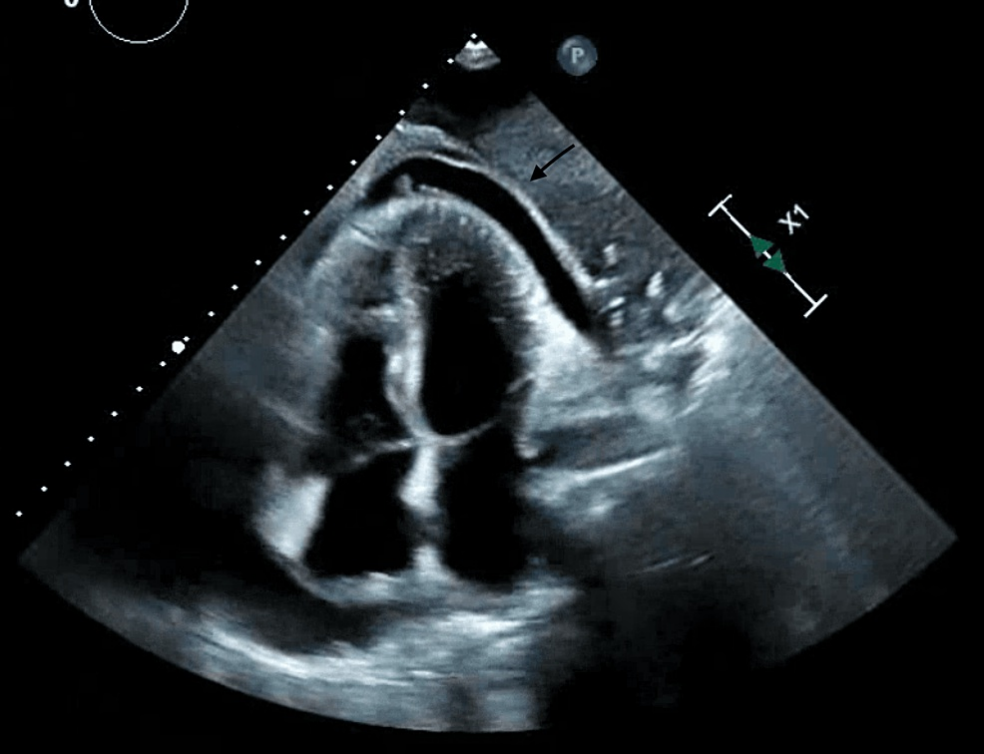
Large pericardial effusion seen on the four-chamber view of echocardiogram.

Her echocardiogram showed a left ventricle ejection fraction (LVEF) of 65 to 70%, with moderate to large pericardial effusion best appreciated in Figure 3. No tamponade physiology was noted.

Blood cultures grew *S. pneumoniae*

Subsequently, antibiotics were deescalated to IV ceftriaxone. She underwent pericardiocentesis and pericardial drain placement, draining 360 mL of serosanguinous fluid. Pericardial fluid analysis showed a white blood cell count (WBC) of 3,725/μL with a neutrophilic shift, and cultures showed no growth. She also underwent CT-guided left chest tube placement. Pleural fluid analysis showed an exudative effusion with a WBC of 20,772/μL. Fluid culture, including fungal, bacterial, and acid-fast bacteria, remained negative. Pleural and pericardial fluid analyses can be seen in Tables 1-2.

**Table 1:** Exudative pericardial and pleural fluid with a neutrophilic shift.

	Pleural	Pericardial	Reference range
Character	Cloudy	Cloudy	
Color	Yellow	Red	
WBC	20,772	3,725	No established reference ranges available for these type of body fluids
RBC	1,925	50,800	No established reference ranges available for these type of body fluids
Neutrophils	95%	88%	No established reference ranges available for these type of body fluids
Mononuclear cells	5%	12%	No established reference ranges available for these type of body fluids

**Table 2:** Exudative pleural and pericardial fluid analysis.

	Pleural fluid	Pericardial fluid	Blood	Reference value
Amylase	13 U/L	13 U/L		No established reference range available for these type of body fluids
Glucose	<10 mg/dL	<10 mg/dL		No established reference range available for these type of body fluids
LDH	3,239 U/L	2,483 U/L		No established reference range available for these type of body fluids
Protein	3.2 g/dL	3.1 g/dL	3.4 g/dL (reference range: 6.0-8.0 g/dL)	No established reference range available for these type of body fluids
Triglyceride	78 mg/dL	101 mg/dL		No established reference range available for these type of body fluids
PH	7	8		No established reference range available for these type of body fluids

Multiple intracavitary lytic therapies were performed through the chest tube due to the loculated nature of the pleural effusion seen on CT. Furthermore, the patient required intubation for acute hypoxic respiratory failure, with increasing vasopressor requirements. A repeat CT imaging of her chest showed necrotizing pneumonia with parenchymal cavitation, but her pleural and pericardial effusion showed improvement, leading to chest tube and pericardial drain removal. Serial echocardiograms showed no re-accumulation of fluid within the pericardial space. Slow clinical improvement was observed, and the patient was successfully extubated, with gradual weaning off pressors.

**Figure 4:**
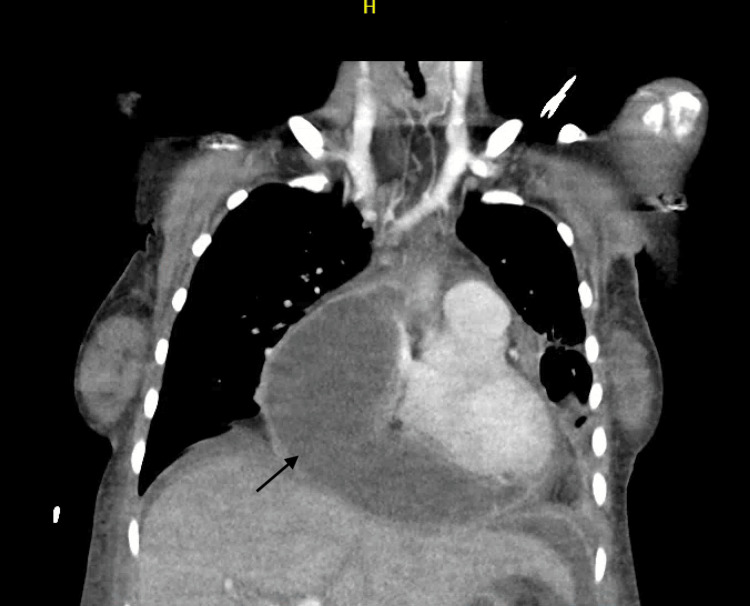
Pericardial abscess seen on the coronal section of chest CT scan.

On day 21 of admission, the patient started spiking fevers. A bedside echocardiogram showed a density in the right atrium with an unchanged EF. Further investigation with a TEE showed no vegetation on any cardiac valve. However, a large cystic space adjacent to and compressing the right atrium, rendering it tubular in shape, was observed. The cystic space contained extensive fibrinous debris, appearing to be in the pericardial space, measuring approximately 8 cm × 8 cm, likely a pericardial abscess, which can be seen in Figure 4.

The patient underwent urgent pericardial abscess evacuation with partial pericardiectomy and pericardial window creation via a subxiphoid approach. The abscess cavity was evacuated and irrigated with antibiotics. No frank pus was observed, but a significant amount of fibrinous material and serosanguinous fluid was drained. Fluid analysis showed a WBC of 33,500/μL with a neutrophilic shift, and the culture showed no growth.

The patient demonstrated significant clinical improvement and was discharged on Cefpodoxime and Metronidazole for three weeks. Follow-up CT chest imaging four weeks post-discharge showed similar chronic changes in the lungs with marked clinical improvement in the patient's functional status. A repeat echocardiogram in six weeks was recommended.

## Discussion

Purulent pericarditis is defined as a confined infection within the pericardial space. It was reported to be three times more frequent in the pre-antibiotic era. With the advent of antibiotics, the incidence of purulent pericarditis has decreased tremendously [2].

Multiple mechanisms have been described in the pathophysiology of purulent pericarditis, including direct spread from an intra-thoracic focus of infection (e.g., pneumonia), hematogenous spread, extension from a myocardial focus (e.g., rupture of periventricular access in patients with endocarditis), extension from a sub-diaphragmatic suppurative focus, or direct infection from trauma, cardio, or thoracic surgery [3].

In the pre-antibiotic era, *S. pneumoniae* accounted for the most common cause of purulent pericarditis. With the introduction of antibiotics and the pneumococcal conjugate vaccine, the incidence decreased from 51% of all cases to 9% [4]. Cardiac and thoracic surgeries currently remain the most common risk factors in immunocompetent patients [2].

In our case, we believe community-acquired pneumonia and *S. pneumoniae* bacteremia were the most likely sources of infection, causing a contiguous spread to the pericardial space. The negative culture from the drained pericardial and pleural fluid could be explained by the early initiation of broad-spectrum antibiotics in the setting of the patient’s septic shock presentation. Several case reports were found in the literature reporting pneumococcal pneumonia complicated with purulent pericarditis and tamponade [5]. However, none of them had evidence of a pericardial abscess.

High clinical suspicion is evidently important due to the low incidence of purulent pericarditis, in addition to the variable clinical features patients may present with. Classic manifestations of pericarditis include fever, which is the most common presentation. Chest pain, which could be pleuritic or non-pleuritic, may occur in 25-37% of cases. Pericardial friction rub occurs in 35-45% of cases, while cardiac tamponade is reported in 42-77% of acute pericarditis cases. [2]

The echocardiogram has a high degree of sensitivity and specificity in detecting pericardial effusion; it also helps identify cardiac tamponade, which is a fatal complication of acute pericarditis. Echocardiography can confirm the diagnosis of tamponade by revealing a moderately large or significant circumferential pericardial effusion. Additionally, it can demonstrate right atrial compression, abnormal respiratory variation in the dimensions of the right and left ventricles, as well as changes in tricuspid and mitral valve flow velocities in most cases. However, it cannot detect the difference between purulent effusion and the effusion of other mechanisms. Regardless, it remains the primary diagnostic tool of choice for suspected pericardial effusion [6].

The mainstay treatments for purulent pericarditis include pericardial drainage and antibiotic therapy. Multiple techniques are described for pericardial drainage, including pericardiocentesis, which is the preferred technique as it provides both diagnostic and therapeutic value. Other techniques include subxiphoid pericardiotomy and pericardiectomy. These interventions are especially used in the case of thick, purulent drainage, loculation, or fibrin depositions. They allow better drainage and prevent the progression of constrictive pericarditis [7]. Antibiotic therapy is just as important. A specific antimicrobial agent according to the causative bacteria identified is indicated [7].

In our unique case report, the patient initially demonstrated improvement, yet despite undergoing surgery and receiving appropriate antibiotic treatment, her condition deteriorated with an escalating need for vasopressors and worsening hypoxic respiratory failure, which raised our concern for the development of cardiac tamponade, the cause of death for patients with bacterial pericarditis [8].

This highlights the importance of a bedside echocardiogram, which would provide us with limited but critical information regarding the hemodynamic status of the patient. Having high clinical suspicion for cardiac tamponade should prompt the utilization of an echocardiogram as the primary diagnostic tool, as it would elicit a rapid response in the management of this cardiac emergency; nonetheless, it improves the chance of recovery and survival [9].

## Conclusions

Purulent pericarditis due to pneumococcal pneumonia is now rare due to antibiotics and pneumococcal vaccines. Early diagnosis and vigilant clinical suspicion are crucial in preventing life-threatening complications such as cardiac tamponade. Bedside echocardiograms aid in assessing patients' hemodynamic status and guiding medical management. Timely antibiotic therapy and surgical intervention significantly reduce mortality rates and prevent complications like constrictive pericarditis.
